# The mouse model of fragile X syndrome exhibits deficits in contagious itch behavior

**DOI:** 10.1038/s41598-020-72891-x

**Published:** 2020-10-19

**Authors:** Rodrigo Gonzales-Rojas, Amtul-Noor Rana, Peter Mason, Christopher Renfro, Vallabhi Annaluru, Shree Panda, Hye Young Lee

**Affiliations:** The Department of Cellular and Integrative Physiology, The University of Texas Health Science Center at San Antonio, San Antonio, TX USA

**Keywords:** Behavioural methods, Autism spectrum disorders

## Abstract

Individuals with autism spectrum disorders (ASDs) imitate observed behavior less than age-matched and typically developing peers, resulting in deterred learning ability and social interaction. However, this deficit lacks preclinical assessment tools. A previous study has shown that mice exhibit contagious itch behavior while viewing a scratching demonstrator mouse, as opposed to an ambulating demonstrator mouse, but whether autism mouse models imitate observed scratching behavior remains unknown. Here, we investigated contagious itch behavior in the mouse model of fragile X syndrome (FXS), a common form of inherited intellectual disabilities with a high risk for ASDs. We found that the mouse model of FXS shows deficits in contagious itch behavior. Our findings can be used as a new preclinical assessment tool for measuring imitative deficits in the study of neurodevelopmental disorders including FXS.

## Introduction

Throughout life, imitative deficits characterize the behavioral manifestation of neurodevelopmental disorders such as autism spectrum disorders (ASDs), especially during early development where imitation facilitates learning and social interaction^1^. In learning contexts, imitative deficits are shown by impairments in vocalization, object use, body movement, and facial replication^2–4^. In socially interactive contexts, children with ASDs fail to imitate contagious behaviors, such as yawning, at the rates of their age-matched and typically developing peers^5^. As a consequence, the impaired imitative ability of children with ASDs fundamentally deters their ability to learn and socially interact^6^. Contagious behaviors can also evoke mirroring responses in socially complex animals, such as primates or dogs^7–9^. Notably, Yu et al. claimed that mice also possess the social complexity to imitate contagious itch behavior^10^. Specifically, mice observing a scratching demonstrator mouse—both with olfactory cues (adjacent home cage paradigm) and without olfactory cues (screen paradigm)—imitated the observed scratching behavior, as opposed to mice observing an ambulating demonstrator mouse. This finding implicates that mice can recapitulate a degree of imitative behaviors seen in humans and other socially complex animals. However, imitative deficits shown in ASD patients have yet to be demonstrated in autism mouse models.

Fragile X syndrome (FXS) is a common form of inherited intellectual disabilities with a high risk for ASDs and is caused by a loss-of-function mutation in the fragile X mental retardation 1 (*FMR1*) gene^11^. While FXS and ASDs are separate clinical entities, sixty percent of males with FXS also show autistic symptoms^12^. Children affected by FXS with autistic symptoms have shown significant impairment in non-meaningful imitative tasks, when compared to children affected by FXS without autistic symptoms^13^. As various autism mouse models recapitulate behaviors associated with human patients, *Fmr1* KO mice, the mouse model of FXS, also exhibit autistic-like behaviors^14^, making them excellent preclinical models for studying FXS. Therefore, the development of a novel assessment to measure imitative deficits using autism mouse models would greatly expand evaluative tools.

Given that mice exhibit visually transmitted contagious itch behavior^10^, we investigated whether the mouse model of FXS shows deficits in imitative scratching behaviors. Here, we found that wild-type (WT) mice displayed imitative scratching behavior when exposed to a scratching demonstrator on a monitor screen while *Fmr1* KO mice failed to imitate despite the fact that no significant differences in spontaneous scratching, look, and self-grooming behaviors were found. Additionally, we did not observe imitative scratching behavior in a group of mice watching an ambulating demonstrator, suggesting that contagious itch behavior was purely induced by watching a scratching demonstrator. Our findings that *Fmr1* KO mice demonstrate impaired contagious itch behavior can provide a novel assessment for imitative deficits, and contribute to the understanding of neurodevelopmental disorders, especially FXS.

## Methods and materials

### Animal care and use

WT (FVB.129P2-*Pde6b*^+^*Tyr*^*c-ch*^/AntJ) and *Fmr1* KO (FVB.129P2-*Pde6b*^+^*Tyr*^*c-ch*^* Fmr1*^*tm1Cgr*^/J) mice were obtained from Jackson Laboratory. The animals were housed in a room with regular lighting conditions (brightness of 300 lx), which translated to a brightness of 20 lx inside a home cage in a colony rack at a mouse’s eye-level. All mice used in this study are males aged P45–P130. The WT-Scr., *Fmr1* KO-Scr., WT-Amb., *Fmr1* KO-Amb., WT-Emp., and *Fmr1* KO-Emp. had a sample size of 26, 20, 18, 17, 9, and 4, respectively. More detailed numbers can be found in the figure legends. All the experimental protocols were approved by and conducted in accordance with the University of Texas Health Science Center at San Antonio Institutional Animal Care and Use Committee.

### Generating videos of demonstrator mice and empty cylinder

A glass cylinder (1000 mL Pyrex^®^ beaker: 10.8 cm W × 15.8 cm H) filled with 50 mL of bedding was placed in front of a white backdrop with additional lighting overhead. After placing a recording camera directly in front of the cylinder, a short 10 s clip was taken of this condition to create an empty cylinder movie. Without moving the camera, a WT mouse was brought into the recording room and placed inside the glass cylinder with a translucent cover to prevent escape. A 60 min recording was obtained, and it was scored to identify examples of a scratching behavior. This entire process was repeated until an ideal scratching bout was identified. Then within the same video, a sequence of an ambulating behavior was also selected. These videos were re-sized so that the cylinder and the demonstrator mouse on-screen matched the actual sizes of the cylinder and observer mice. Using Movie Studio Platinum 15.0, a video editing software, all three clips (scratching demonstrator, ambulating demonstrator, and empty cylinder) were played on loop for 60 min, alternating between forwards and backwards playback to eliminate abrupt changes of the mouse’s movement. An 11 min black screen was added to the beginning of all videos to accommodate a 1 min setup time and 10 min habituation time for the observer mice. These three videos were shown to WT and *Fmr1* KO mice.

### Look-and-scratch behavior test

The mice were habituated as a group for 60 min with food and water in their home cage (30 cm W × 19 cm L × 13 cm H) in the testing room. The room brightness of the group habitation area was 420 lx, and the brightness inside the home cage was 50 lx at a mouse’s eye-level. Then, the mice were individually placed in a new clean cage (30 cm W × 19 cm L × 13 cm H) with water but no food for 70 min for single cage habituation. The room brightness for the single habituation area was 450 lx, and the brightness inside the single cage was 30 lx at a mouse’s eye level. The mice were then placed in a glass cylinder (1000 mL Pyrex^®^ beaker: 10.8 cm W × 15.8 cm H) with 50 mL of bedding inside, located on a stand matching the elevation of the display monitor. White walls surrounding the recording area prevented distraction, and a lid over the cylinder prevented the mice from escaping. The mice habituated in the cylinder for 10 min. A scratching demonstrator video was then shown for 60 min on-screen for the experimental group. An ambulating demonstrator video with no additional behaviors was shown on-screen for the control group. An empty cylinder video with no mouse was shown on-screen for look behavior analysis and additional quality control. The brightness of the recording room was 530 lx, and the brightness inside the recording cylinder was 210 lx at a mouse’s eye level. The observer mouse was recorded from two camera angles, one being used for analysis purposes and the other for redundancy purposes as shown in Supplementary Fig. S1. The front view of what the mouse is watching is also demonstrated in Supplementary Fig. S1a. The total number of looks, scratching, and self-grooming bouts were analyzed and quantified by 3 different investigators who were blind to the genotype and the video being observed by the test subject. Specifically, behaviors were scored with recordings from the analysis camera angle in order to blind the investigator from the type of video shown to the observer mice.

### Look behavior analysis

The total number of looks was scored by investigators who were blind to the genotype of the observer mice and the videos the mice watched. Look behaviors were defined as a pause and glance towards the screen, as previously described^10^. Sniffing towards the screen within the frame of the demonstrator mouse was also counted as a look. If the mouse was performing any other behaviors such as self-grooming, digging, climbing, or jumping towards the screen, it was not scored as a look unless an obvious deviation from its behavior with a slight glance towards the screen was observed.

### Scratching behavior analysis

The total number of scratching bouts was scored by investigators who were blind to the genotype of the observer mice and the videos the mice watched. The beginning of a scratching bout was defined as a lifting of the hind limb to stroke towards the nape, head, body, or torso; the end of the scratching bout was defined as the lowering of the limb back to the floor, regardless of how many scratching strokes or pauses take place in between, as previously defined^10^. Imitative scratching bouts were defined as a look behavior followed by a scratching bout that starts within the maximum latency of 5 s, as previously described^10^. Spontaneous scratching bouts were defined as a scratching bout with no associated look behavior 5 s prior to the first lifting of the paw. The beginning of each scratching bout was compared to quantified look data to differentiate imitative scratching bouts from spontaneous scratching bouts.

### Self-grooming behavior analysis

The total number of self-grooming bouts was scored by investigators who were blind to the genotype of the observer mice and the videos the mice watched. An individual self-grooming bout was defined as a continuous series of self-grooming chain patterns which include elliptical strokes tightly around the nose, unilateral strokes around the vibrissae, bilateral strokes made by both paws simultaneously, stroking the head and around the ears, and obvious body licking, as previously defined^15^. Self-grooming paw usage is limited to the front paws only. Incomplete or interrupted self-grooming chains were still scored as a single bout as long as the self-grooming behavior resumed within the maximum latency of 6 s, as previously described^15^.

### Statistical analyses

All statistical analyses were performed on GraphPad PRISM. Outliers were identified for each data set within each graph using the ROUT method; the strictest setting of Q = 0.1% was consistently used to only remove definite outliers. Statistical analyses of parametric data sets were performed with a two-way ANOVA or unpaired t test method. Statistical analyses of nonparametric data sets were performed in two steps: first with a Mann–Whitney *U* test corrected for ties, then followed by adjusting the P values for multiple comparisons with the Holm–Sidak test. The test(s) used for each graph and the threshold(s) for significance were indicated in the figure legends.

## Results

### *Fmr1* KO mice show deficits in imitative scratching behavior

We first explored if the mouse model of FXS shows imitative deficits by placing WT or *Fmr1* KO mice (P45–P130) in a clear cylinder in front of a monitor displaying a demonstrator mouse, either scratching or ambulating (Supplementary Fig. S1a), as previously described^10^. We recorded WT and *Fmr1* KO observer mice from two camera angles. The first camera angle was used for analysis, not only because the monitor’s pixilation blinded the viewer from the on-screen demonstrator, but also because it served as a reflective surface on which look behavior could be verified based on the angle of pixilation (Supplementary Fig. S1b). However, from the observer mouse’s perspective, the computer monitor’s pixilation did not create a reflective surface (Supplementary Fig. S1a). The second camera angle served for redundancy so that the on-screen demonstrator (scratching or ambulating) could be verified by the viewer after the completion of analysis (Supplementary Fig. S1c). A scratching bout was defined as the lifting of a hind limb to stroke towards the nape, head, body, or torso of the mouse, and ending with the lowering of that limb back to the floor, regardless of how many strokes or pauses occurred between the initial lifting and final lowering, as previously described^10^. Look behaviors were defined as a pause and glance towards the demonstrator, and imitative scratching bouts were defined as a look behavior followed by a scratching bout within a 5 s latency (look-and-scratch), both as previously described^10^. Because the brightness conditions of experimental settings play an important role in the visual skills of *Fmr1* KO mice^16^, we measured the luminance of various locations where the experiment was conducted. More details on brightness conditions can be found in the “Methods and materials”.

**Figure 1:**
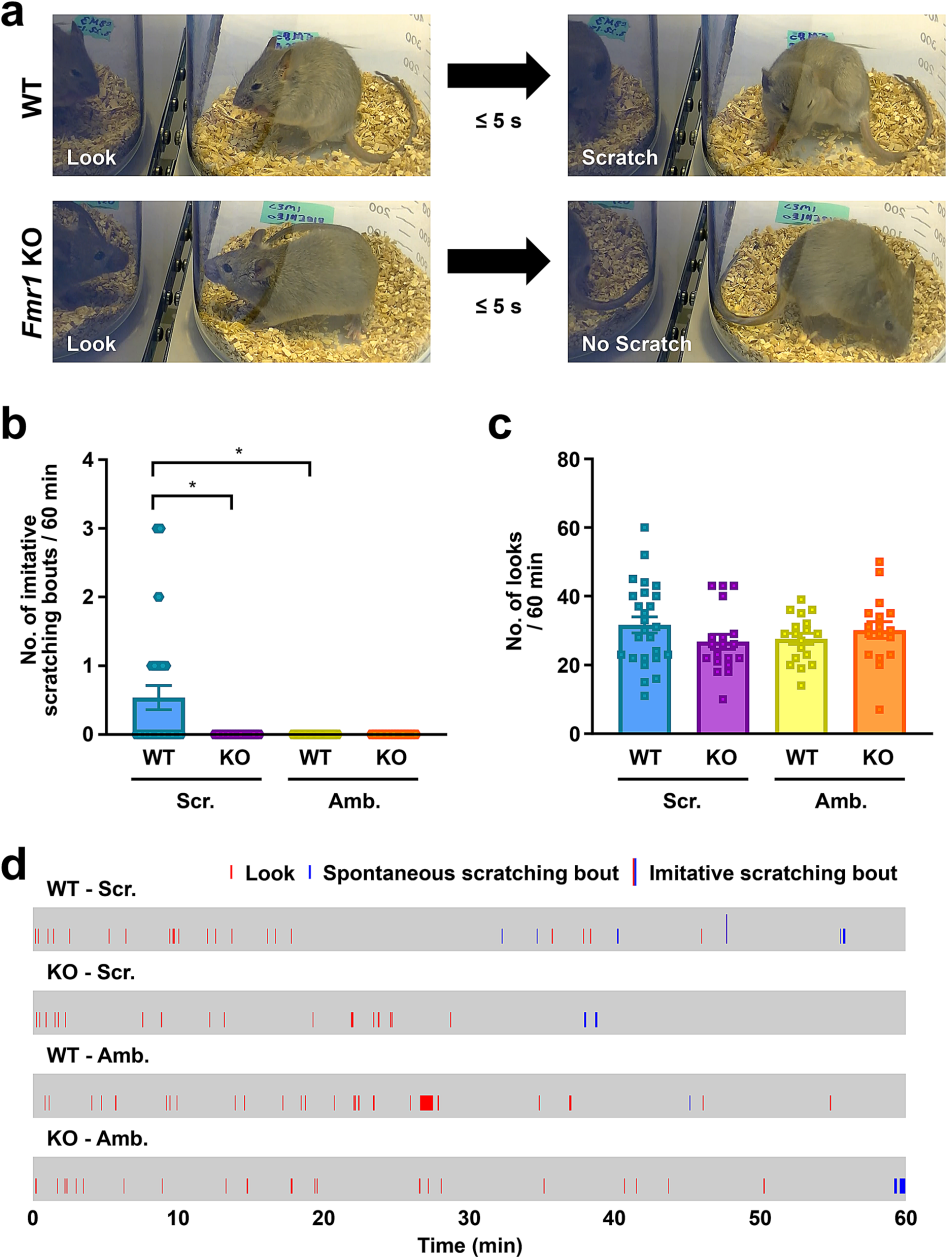
*Fmr1* KO mice exhibit deficits in imitative scratching behavior. (**a**) Representative screenshots of male WT and *Fmr1* KO mice each observing a scratching demonstrator mouse video. The WT mouse starts an imitative scratching bout within 5 s of a look behavior. The *Fmr1* KO mouse fails to demonstrate an imitative scratching bout within the maximum latency of 5 s. (**b**,**c**) The mean number of imitative scratching bouts (**b**) and the mean number of look behaviors (**c**) by WT and *Fmr1* KO mice observers watching a scratching (Scr.) or ambulating (Amb.) demonstrator mouse video. n = 17–26 for each group, mean ± SEM, *P < 0.05, P values were calculated between WT-Scr. and *Fmr1* KO-Scr., WT-Amb. and *Fmr1* KO-Amb., WT-Scr. and WT-Amb., or *Fmr1* KO-Scr. and *Fmr1* KO-Amb. using Mann–Whitney *U* test for nonparametric data (**b**) and two-way ANOVA for parametric data (**c**). (**d**) Representative raster plots for look behaviors, spontaneous scratching bouts, and imitative scratching bouts of WT and *Fmr1* KO mice observers watching a scratching (Scr.) or ambulating (Amb.) demonstrator mouse video.

In response to viewing an on-screen scratching demonstrator, WT mice imitatively scratched, while *Fmr1* KO mice failed to scratch within 5 s after looking towards the screen (representative images are shown in Fig. 1a, and a representative movie is shown in Movie S1 with the two camera angles previously mentioned). Specifically, WT mice watching a scratching demonstrator exhibited an average of approximately 0.54 imitative scratching bouts over 60 min; however, *Fmr1* KO mice watching the same scratching demonstrator failed to exhibit any contagious itch behavior over the same period (Fig. 1b). Importantly, the average number of look behaviors observed in WT (approximately 31.6 looks/60 min) and *Fmr1* KO mice (approximately 26.7 looks/60 min) watching a scratching demonstrator video was not significantly different (Fig. 1c). This suggests that *Fmr1* KO mice fail to imitate scratching behavior independently from look behavior frequency. Furthermore, both WT and *Fmr1* KO mice observing an ambulating demonstrator did not exhibit imitative scratching bouts (Fig. 1b), similar to findings in WT mice reported by Yu et al*.*^10^. This implicates that contagious itch behavior was specifically induced by watching a scratching demonstrator. Moreover, the average number of look behaviors for WT (approximately 27.6 looks/60 min) and *Fmr1* KO mice (approximately 30.1 looks/60 min) watching an ambulating demonstrator was similar between genotypes and was not significantly different from mice watching a scratching demonstrator (Fig. 1c). This confirms that the lack of imitative scratching behavior in mice watching the ambulating demonstrator video was independent from look behavior frequency. Representative raster plots including look behaviors and scratching bouts that occurred during these 60 min of demonstrator exposure are shown in Fig. 1d. Taken together, our results demonstrate that WT mice can imitatively scratch while the mouse model of FXS fails to imitate, despite that no significant differences in look behavior between genotypes were found.

### *Fmr1* KO mice demonstrate normal spontaneous scratching, look, and self-grooming behaviors

**Figure 2:**
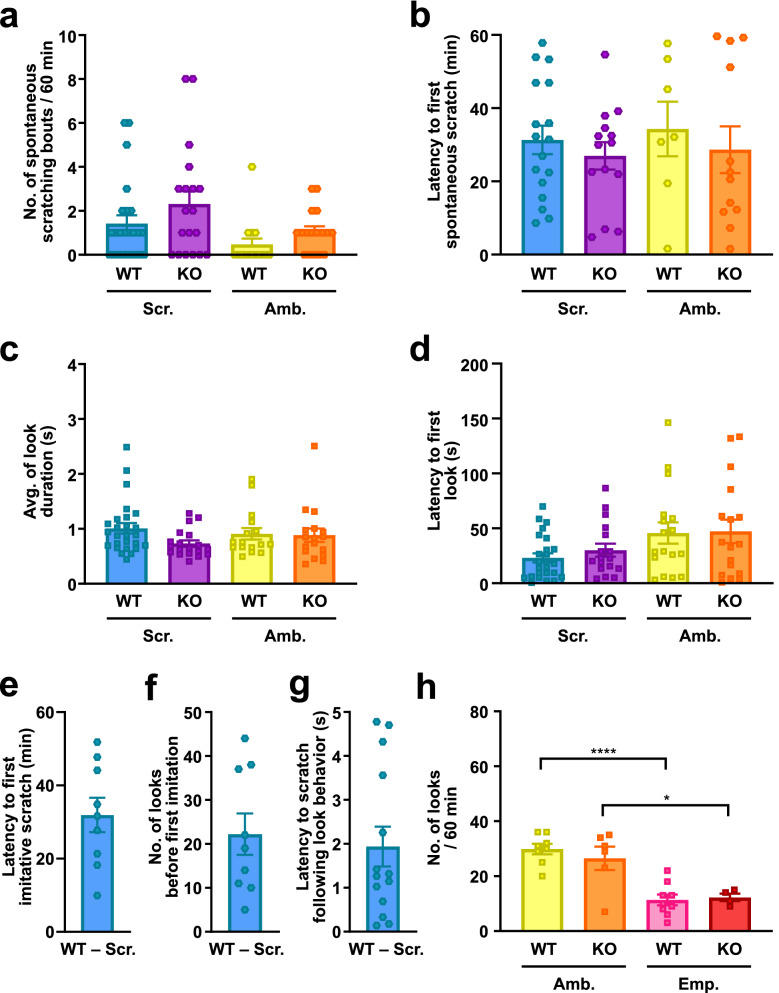
*Fmr1* KO mice demonstrate normal spontaneous scratching and look behaviors. (**a**–**d**) Mean number of spontaneous scratching bouts (**a**), latency to the first spontaneous scratching behavior (**b**), mean duration of look behavior (**c**), and latency to the first look behavior (**d**) by WT and *Fmr1* KO mice observers watching a scratching (Scr.) or ambulating (Amb.) demonstrator mouse video. n = 7–26 for each group, mean ± SEM, P values were calculated between WT-Scr. and *Fmr1* KO-Scr., WT-Amb. and *Fmr1* KO-Amb., WT-Scr. and WT-Amb., or *Fmr1* KO-Scr. and *Fmr1* KO-Amb. using Mann–Whitney *U* test for nonparametric data (**a**) and two-way ANOVA for parametric data (**b**–**d**). (**e–g**) Latency to the first imitative scratching behavior (**e**), mean number of looks prior to performing an imitative scratching bout (**f**), and latency to an imitative scratching bout following a look behavior (**g**) by WT mice observers watching a scratching (Scr.) demonstrator mouse video. n = 9–14 for each group, mean ± SEM. (**h**) The mean number of look behaviors by WT and *Fmr1* KO mice observers towards an ambulating (Amb.) demonstrator mouse or empty cylinder (Emp.) video. n = 4–9 for each group, mean ± SEM, *P < 0.05, ****P < 0.0001, P values were calculated between WT-Amb. and *Fmr1* KO-Amb., WT-Emp. and *Fmr1* KO-Emp., WT-Amb. and WT-Emp., or *Fmr1* KO-Amb. and *Fmr1* KO-Emp. using two-way ANOVA.

Next, we examined both the number of spontaneous scratching bouts—defined as the total number of scratching bouts, excluding imitative scratching bouts—(Fig. 2a) and the latency to exhibit the first spontaneous scratching bout (Fig. 2b) in WT and *Fmr1* KO mice viewing a scratching or ambulating demonstrator. As a result, mice viewing a scratching demonstrator showed no significant differences between genotypes in both the number and latency. In addition, within the same genotype, mice watching a scratching or ambulating demonstrator also showed no significant differences in both the number and latency. This suggests that the imitative scratching deficit shown in *Fmr1* KO mice is independent from spontaneous scratching behaviors (frequency and latency). The average look duration (Fig. 2c) and the latency to exhibit the first look behavior (Fig. 2d) for both WT and *Fmr1* KO mice viewing a scratching or ambulating demonstrator showed no significant differences across all cohorts. This implicates that the imitative scratching deficit in *Fmr1* KO mice was not caused by the levels of visual attention given to the demonstrator video. The first imitative scratching bout in WT mice watching a scratching demonstrator video appeared after approximately 31.9 min (Fig. 2e) and approximately 22.2 looks (Fig. 2f). The latency to an imitative scratching bout following a look behavior was approximately 1.9 s (Fig. 2g), close to the latency (approximately 2.2 s) reported by Yu et al*.*^10^ Notably, we verified from Yu et al. that look behavior towards the monitor screen is not random, and can be induced by the presence of a mouse^10^. Specifically, we found a significant decrease in the number of looks in both WT and *Fmr1* KO mice watching an ambulating demonstrator versus an empty cylinder video (Fig. 2h). Lastly, to test whether *Fmr1* KO mice showed other behavioral changes, we measured self-grooming behaviors during our contagious itch assay. As a result, we found that WT and *Fmr1* KO mice viewing a scratching or ambulating demonstrator video showed no significance differences in the total number of self-grooming bouts (Supplementary Fig. S2a), mean duration per self-grooming bout (Supplementary Fig. S2b), and total self-grooming duration (Supplementary Fig. S2c). Taken together, our results implicate that *Fmr1* KO mice fail to produce contagious itch behavior, independently from spontaneous scratching, look, and self-grooming behaviors.

### Age differences do not impact imitative scratching, look, or spontaneous scratching behaviors

In our study, we used male mice ranging P45–P130 to include the cohort ages used by Yu et al*.* (P42–P84) and Lu et al*.* (P42–P56) who performed similar assays that assessed for imitative scratching behaviors in mice^10,17^. Notably, previous studies have defined these age ranges as mid-late adolescence to adulthood^18,19^. Given that our experiment replicated similar conditions to Yu et al*.*, we attempted to encapsulate similar age groups (mid-late adolescence and adulthood) as them while also covering the age groups used by Lu et al*.* To explore whether differences in these two age groups affected behavioral assessments performed in our study, we separated the results of imitative scratching, look, and spontaneous scratching behaviors from the WT group watching a scratching demonstrator (Fig. 1b,c, 2a,b,d,e) into adolescent (P45–P59) and adult (P60–P130) cohorts (Supplementary Fig. S3). As a result, we found no significant differences in the total number of behaviors as well as the latency to the first behaviors between adolescent and adult WT groups (Supplementary Fig. S3). Taken together, these comparisons suggest that the differences in age groups did not affect look or scratching behaviors.

## Discussion

In this study, we confirmed the presence of contagious itch behavior in mice^10^. We further showed that *Fmr1* KO mice demonstrate a deficit in imitative scratching behavior when compared to WT mice while watching an on-screen scratching demonstrator. The original discovery from Yu et al. that mice exhibit visually transmitted contagious itch has been controversial^10^. Liljencrantz et al. failed to reproduce contagious itch behavior with the adjacent home cages method from Yu et al.^20^ Barry et al. then replied, strongly suggesting the use of the screen paradigm^21^. Later, Lu et al. could not identify contagious itch behavior with a modified version of the screen paradigm from Yu et al., which removed the necessity to quantify look behavior^17^. In our study, we defined an imitative scratching behavior identically to that of Yu et al.: a look or glance towards the screen, and then a scratching bout within 5 s. However, Lu et al. defined an imitative scratching bout as any scratching bout when a mouse was exposed to demonstrator stimulus on four sides horizontally. We argue that defining an imitative scratching bout requires an obvious look prior to scratching, which guarantees that the behavior is not coincidental. Lastly, we recorded observer mice watching demonstrator stimuli for 60 min, as performed by Yu et al., while Lu et al. analyzed observer mice for 30 min, which may contribute to this discrepancy. Notably, we found a smaller average number of imitative scratching bouts compared to Yu et al. This finding can be caused by strain differences between FVB and C57BL/6J backgrounds, given that FVB mice are known to display higher locomotor activities than C57BL/6J mice^22^. Therefore, potential future studies can use our novel assessment to measure deficits in contagious itch behavior in various strains of autism mouse models.

ASD patients show well-documented eye contact deficits in both socially interactive contexts^23^ and during an evaluation of contagious yawning behavior susceptibility^5^. However, in our study, imitative scratching deficits shown in *Fmr1* KO mice are independent from look behavior given the statistical similarity of look behavior frequency and latency to the first look between WT and *Fmr1* KO mice when exposed to a demonstrator mouse. Additionally, the statistical similarity of average look number between WT and *Fmr1* KO mice watching a scratching or ambulating demonstrator was contrasted by a significant decrease in average look number by groups watching an empty cylinder, suggesting that look behavior is not random and is dependent on the presence of a mouse. Notably, FXS patients also show impaired visual contrast discrimination^24,25^, and *Fmr1* KO mice recapitulate this deficit^16,26^. However, we do not believe that this contrast perception deficit played a role in the imitative responses of *Fmr1* KO mice in our study. Specifically, our findings of look behavior towards a gray demonstrator mouse against a white background reproduced a finding from Felgerolle et al*.* where *Fmr1* KO mice showed no visual contrast deficits in a gray against white contrast condition^16^. Notably, different contrast conditions used by Felgerolle et al*.*, such as black against white or light gray against white, showed significantly reduced look behaviors in *Fmr1* KO mice compared to WT mice^16^. Collectively, given our experimental conditions, we conclude that imitative scratching deficits observed in *Fmr1* KO mice in our study are independent of previously reported visual deficits in FXS and *Fmr1* KO mice. Furthermore, other behaviors we examined such as spontaneous scratching and self-grooming behaviors showed no significant differences between genotypes. These results suggest that other types of behaviors that we examined during our contagious itch assay did not affect the results obtained in this study.

Here, we found that *Fmr1* KO mice demonstrate a significant decrease in contagious itch behavior, establishing a novel phenotype in the mouse model for FXS. Our study serves as a promising tool to measure imitative deficits using preclinical mouse models, which recapitulate a subset of social deficits shown in FXS^13^ and ASDs^6^. While contagious behaviors in typically developing humans are arguably driven by empathetic ability^27^, empathetic deficits are a prominent phenotype in ASDs and might give rise to imitative deficits^5^. Although the existence of empathic behavior in WT mice is highly debated, our findings potentially expand this discussion surrounding the presence of empathetic behavior in rodents. We also believe that our findings can lead to exciting future studies that identify brain regions contributing to the deficits in contagious itch behavior shown in *Fmr1* KO mice. Notably, Yu et al. demonstrated that the suprachiasmatic nucleus (SCN) mediates contagious itch behavior^10^, which leads us to question whether this brain region also contributes to imitative behavioral deficits seen in *Fmr1* KO mice in our study. Future works within this realm can also include testing in different strains, ages, or genders of mice. This will further establish the consistency or variance of contagious itch behavior present in WT and *Fmr1* KO mice. Lastly, this novel assessment can be further tested in other autism mouse models to facilitate a greater understanding of neurodevelopmental disorders.

## Supplementary information

Supplementary file1

Supplementary file2

## Data Availability

The authors declare that all data supporting the findings of this study are available within the paper and its Supplementary Information.
