# Supplementary material for: The mouse model of fragile X syndrome exhibits deficits in contagious itch behavior
Source: Sci Rep. 2020 Oct 19;10:17679. doi: 10.1038/s41598-020-72891-x (PMC7573600; doi:10.1038/s41598-020-72891-x)
Supplement: Supplementary file 1 — Supplementary file1 [file 41598_2020_72891_MOESM1_ESM.pdf]

Supplementary Materials for

The mouse model of fragile X syndrome exhibits deficits in contagious itch behavior

Rodrigo Gonzales-Rojas*, Amtul-Noor Rana*, Peter Mason, Christopher Renfro, Vallabhi Annaluru, Shree Panda and Hye Young Lee**

*These authors contributed equally: Rodrigo Gonzales-Rojas and Amtul-Noor Rana
**Correspondence to: Hye Young Lee (Leeh6@uthscsa.edu)

This PDF file includes:

Figure S1-S3
Captions for Movie S1

Other Supplementary Materials for this manuscript include the following:

Movie S1

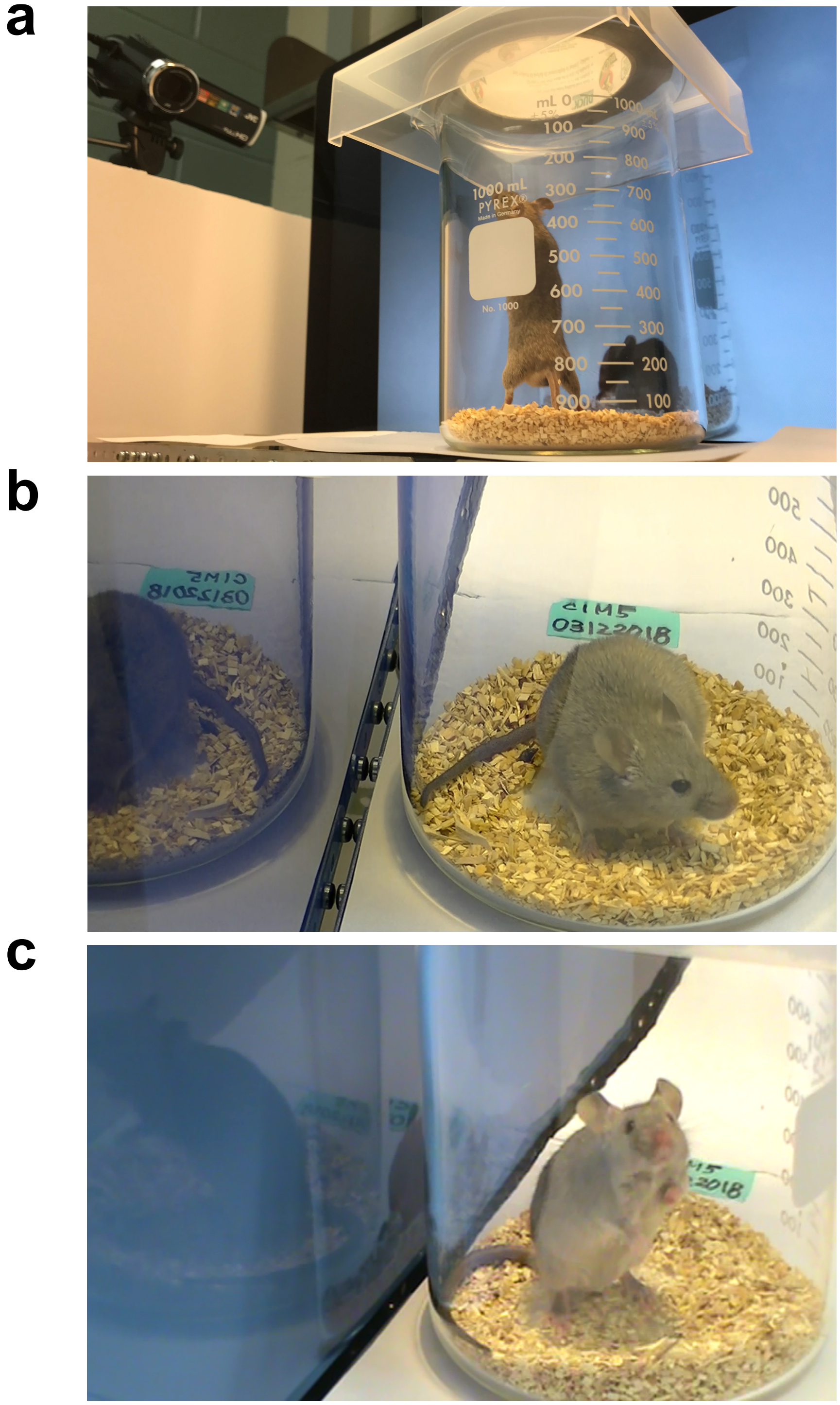

Figure S1. Related to Figure 1.
Recording conditions for mice observers and camera angles for analysis purposes. (**a**) Recording conditions illustrate how the mouse observer was placed on a stand matching the elevation of the screen. The movie was zoomed out so the demonstrator's size matched that of the observer mouse's. (**b**) The first camera angle was used for behavioral analysis from which the demonstrator mouse video watched by the observer mouse is not visible to an experimenter. The reflection of the observer mouse is seen in this angle. (**c**) The second camera angle was used for redundancy from which the demonstrator mouse video watched by the observer mouse is clearly visible to an experimenter.

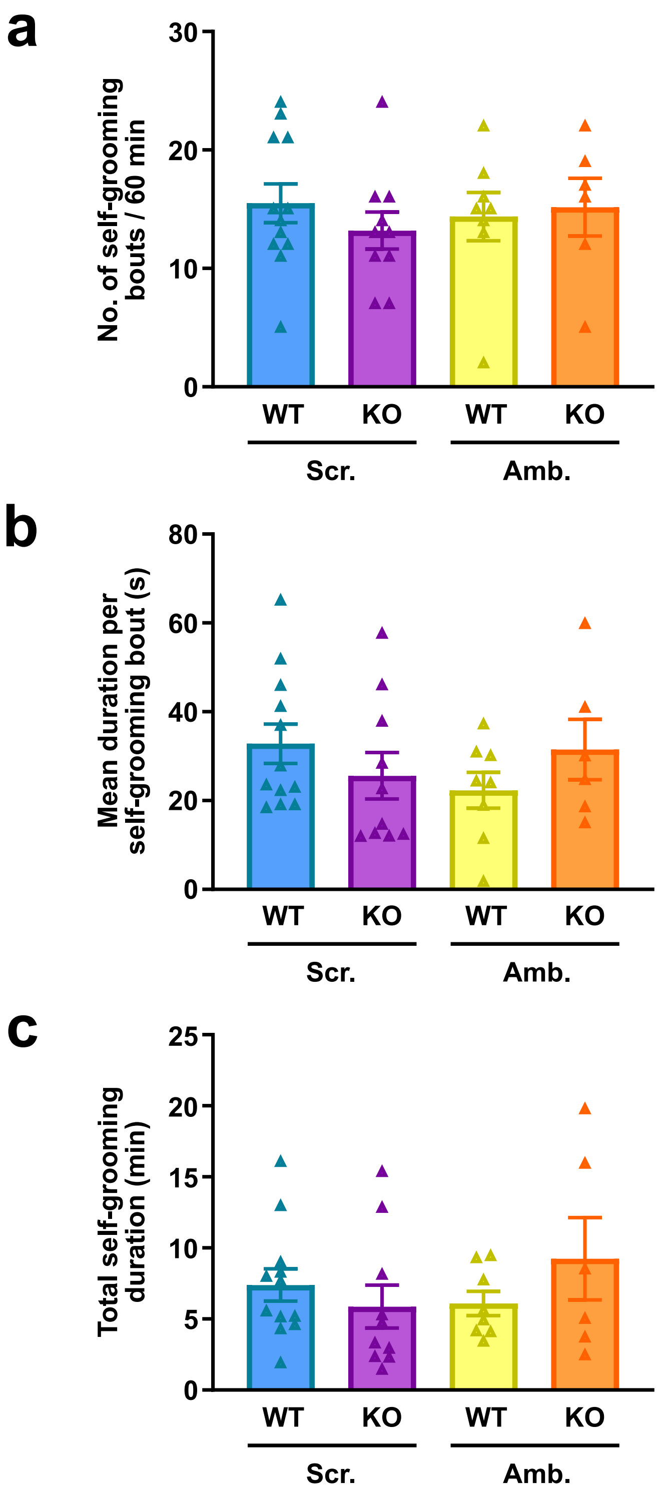

Figure S2. Related to Figure 2.
Fmr1 KO mice show no significant differences in self-grooming behaviors. (**a**–**c**) Total number of self-grooming bouts (**a**), mean duration per self-grooming bout (**b**), and total self-grooming duration (**c**) by WT and *Fmr1* KO mice observers watching a scratching (Scr.) or ambulating (Amb.) demonstrator mouse video. n=7-12 for each group, mean ± SEM, P values were calculated between WT-Scr. and *Fmr1* KO-Scr., WT-Amb. and *Fmr1* KO-Amb., WT-Scr. and WT-Amb., or *Fmr1* KO-Scr. and *Fmr1* KO-Amb. using two-way ANOVA.

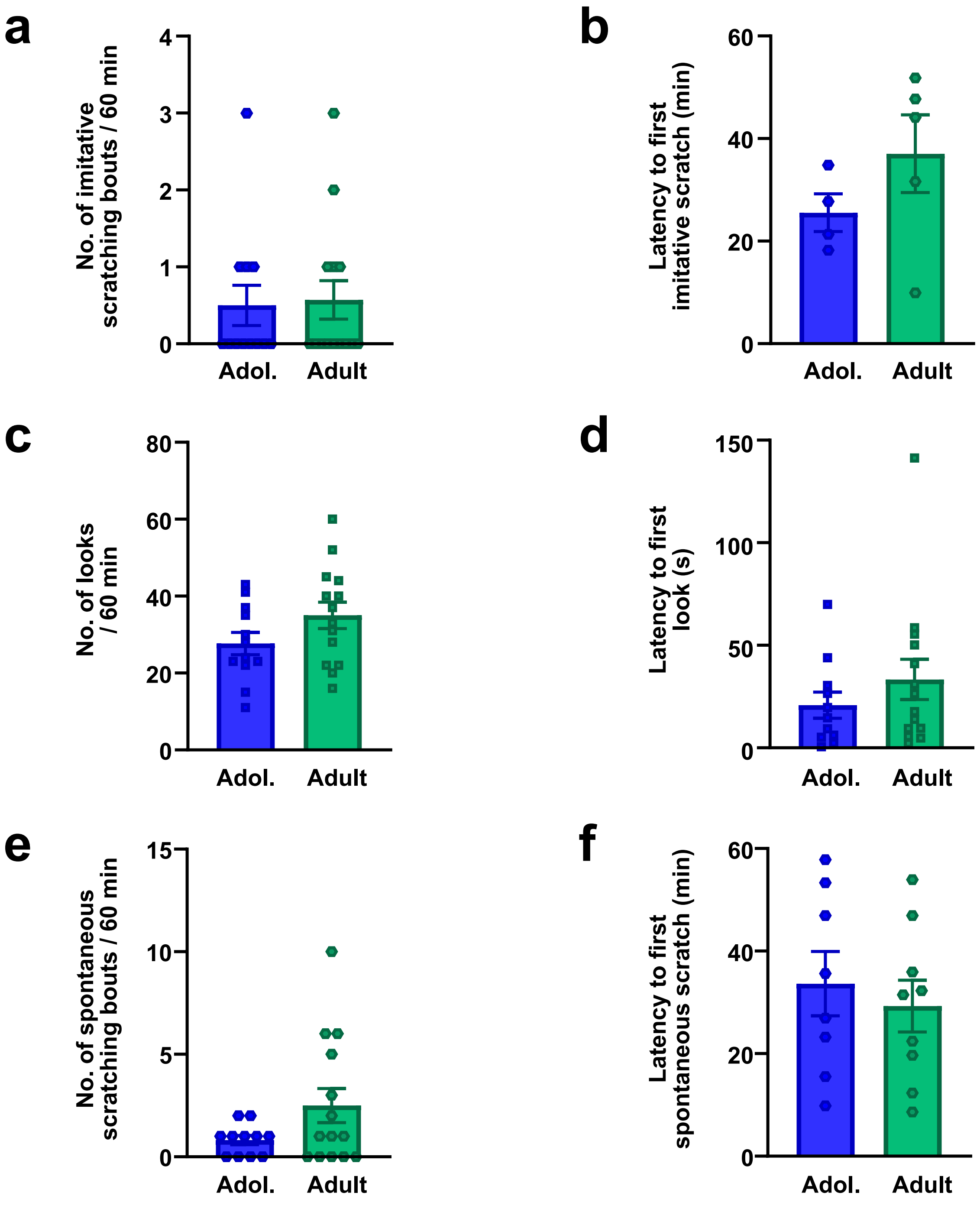

Figure S3. Related to Figures 1 and 2.
Differences in age groups do not impact imitative scratching, look, or spontaneous scratching behaviors. Data from existing graphs in Figures 1 and 2 were separated into mid-late adolescent (P45 – P59) and adult (P60 – P130) groups. (**a**–**f**) Mean number of imitative scratching behaviors (**a**), latency in minutes to the first imitative scratching bout (**b**), mean number of looks (**c**), latency in seconds to the first look behavior (**d**), mean number of spontaneous scratching behaviors (**e**), and latency in minutes to the first spontaneous scratching bout (**f**) between mid-late adolescent (Adol.) and adult (Adult) WT mice observers watching a scratching demonstrator mouse video. (**a**–**f**) n=4-14 for each group, mean ± SEM, P values were calculated between Adol. and Adult. using Mann–Whitney *U* test for nonparametric data (**a, e**) and unpaired t-test for parametric data (**b**–**d, f**).

Movie S1. Related to Figure 1.

Contagious itch deficits in *Fmr1* KO mice. This movie illustrates how a male WT mouse observer (top panel) will look towards an on-screen scratching demonstrator and within 5 s, begins an imitative scratching bout. Secondly, the video demonstrates how a male *Fmr1* KO mouse observer fails to exhibit contagious itch behavior, despite looking towards a scratching stimulus, within the maximum latency of 5 s. This movie also depicts the two separate camera angles from which the observer mice were recorded, the first for analysis purposes, and the second for redundancy from which the demonstrator stimulus can be verified. This video is played at 1X speed, and the software used to edit it are Movie Studio Platinum 15.0 and Microsoft PowerPoint.
